# Efficacy and safety of the vein of Marshall ethanol infusion with radiofrequency catheter ablation for the treatment of persistent atrial fibrillation in elderly patients

**DOI:** 10.3389/fcvm.2023.1276317

**Published:** 2023-12-07

**Authors:** Tao Luo, Yanhong Chen, Xiong Xiong, Guanghui Cheng, Chenggang Deng, Jinlin Zhang

**Affiliations:** Department of Cardiology, Wuhan Asian Heart Hospital, Wuhan, Hubei, China

**Keywords:** catheter ablation, vein of Marshall, persistent atrial fibrillation, elderly patients, efficacy, safety

## Abstract

**Background:**

Increasing age is a significant risk factor for atrial fibrillation (AF) recurrence after catheter ablation (CA). We accomplished this study to evaluate the efficacy and safety of the vein of Marshall (VOM) ethanol infusion (VOM-EI) with CA in elderly patients with persistent AF (PsAF).

**Methods:**

This retrospective observational study included 360 consecutive adult patients with PsAF, of which 141 were in the Elder group (age ≥65 years) and 219 were in the Younger group (age <65 years), who underwent the VOM-EI and radiofrequency CA (RFCA) between May 2020 and April 2022. The efficacy endpoint was no recurrence of AF within one year after CA.

**Results:**

The VOM-EI was successfully performed in 90.8% of patients from the Elder and 88.6% from the Younger group. All patients achieved PVI; 97.9% of patients from the Elder and 98.6% from the Younger group reached LA roof block, and 93.6% of patients from the Elder and 95.9% from the Younger group achieved MI block. There was no significant difference in 1-year survival without recurrence of AF between the two groups (83.0% and 84.5%, respectively). The incidence of complications within 30 days after the procedure from the two groups was low and did not differ significantly.

**Conclusion:**

The VOM-EI combined with RFCA proved to be an effective and safe strategy for treating PsAF in elderly and younger patients.

## 1. Introduction

Atrial fibrillation (AF) is a common tachyarrhythmia in elderly patients, increasing the risk of heart failure and stroke (1). Catheter ablation (CA) has become the most effective strategy for treating AF (2). Current guidelines recommend CA for patients with symptomatic paroxysmal AF or persistent AF (PsAF) refractory to antiarrhythmic drugs (AADs) (3). Pulmonary vein isolation (PVI) can achieve satisfactory clinical results for paroxysmal AF. However, PVI alone is associated with suboptimal outcomes for PsAF, and more expansive ablation is advocated (4–7). Linear and complex fractionated atrial electrogram (CFAE) ablation are widely recommended strategies for atrial substrate modification beyond PVI for treating PsAF (8–10). However, the benefits of adjunctive ablation remain to be determined (11–14). The vein of Marshall (VOM) contains arrhythmogenic foci, myocardial connections, and innervation that can be ablated by ethanol infusion, making it an ideal target in the CA of AF (15). The VOM ethanol infusion (VOM-EI) combined with CA has proven to improve the success rate of CA for PsAF (16, 17). However, increasing age is a significant risk factor for AF recurrence after CA. This retrospective observational study evaluated the efficacy and safety of the VOM-EI with CA in elderly patients with PsAF.

## 2. Materials and methods

### 2.1. Study population

This retrospective observational study included consecutive adult patients with PsAF who underwent the VOM-EI and radiofrequency CA (RFCA) between May 2020 and April 2022. We defined the PsAF as constant episodes of AF beyond seven days and an attack terminated after seven days by cardioversion. Long-standing PsAF was defined as continuous episodes of AF beyond one year when a rhythm control strategy was decided (3). Both PsAF and long-standing PsAF were included in this study. We excluded patients with valvular AF and a history of previous ablation for AF. According to the age of the patients, they were divided into the Elder group (age ≥ 65 years) and the Younger group (age <65 years). The study was approved by the Ethics Committee of Wuhan Asian Heart Hospital (No.2023-B021).

### 2.2. Ablation strategies

The VOM-EI was the initial procedure, followed by RFCA. Circumferential pulmonary vein antrum (CPVA) ablation for achieving PVI and then linear ablation of the left atrium (LA) roof and the mitral isthmus (MI) was performed. If the patient had a history of typical atrial flutter episodes, the cavotricuspid isthmus (CTI) ablation was performed. Activation mapping guided ablation was performed if atrial tachycardia (AT) occurred during the procedure. If AF continued after the above procedures, direct-current cardioversion was performed. PVI and bidirectional block of the ablation line were verified in sinus rhythm. If MI was not blocked, coronary sinus (CS) vein ablation was performed. Complex fractionated atrial electrogram (CFAE) ablation and (or) posterior wall isolation (PWI) were performed at the operator's discretion. The endpoint of ablation was the termination of AF.

### 2.3. Periprocedural management

All patients underwent transesophageal echocardiography immediately before the procedure to exclude thrombus in the LA. Amiodarone was discontinued for at least two weeks, and other AADs for at least five half-lives.

### 2.4. The VOM-EI process

**Figure 1:**
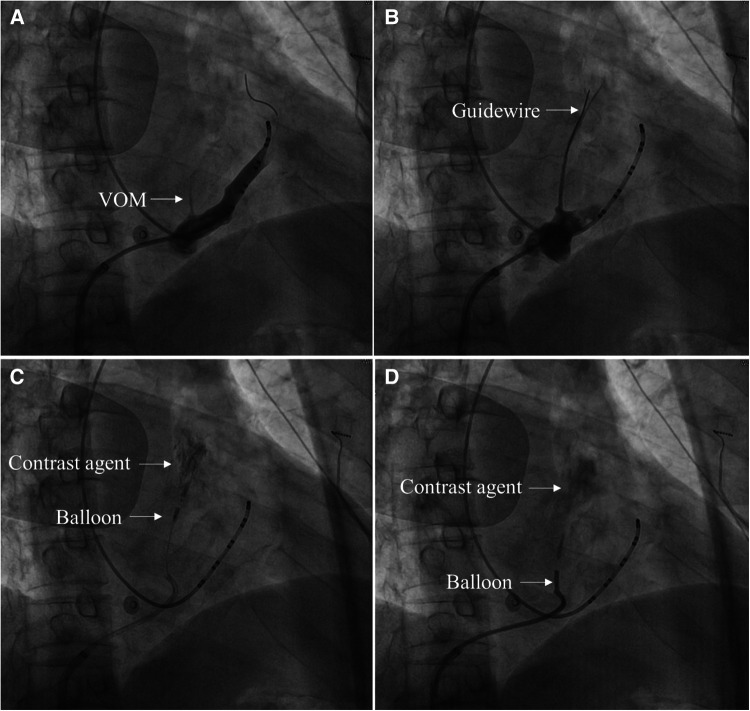
The primary process of the VOM-EI procedure in 30° right anterior oblique views. (**A**) CS vein angiography showed VOM (Arrow indicates the VOM). (**B**) A guidewire was advanced to the distal end of the VOM (Arrow indicates the guidewire). (**C**) After the balloon was dilated at the distal end of the VOM and injected with anhydrous ethanol, local myocardial staining was observed (Arrow indicates the dilated balloon at the distal end of the VOM and the myocardium stained with a contrast agent, respectively). (**D**) After the balloon was dilated at the proximal end of the VOM and injected with anhydrous ethanol, local myocardial staining was observed (Arrow indicates the dilated balloon at the proximal end of the VOM and the myocardium stained with a contrast agent, respectively).

All patients underwent general anesthesia, and the bispectral index monitored the level of sedation and state of consciousness. Intravenous heparin maintained an active coagulation time of 300–350 s during the procedure. A typical protocol of the VOM-EI has been described previously (18); the primary process was:

2.4.1 A 6F JR 3.5 guiding catheter was advanced to the CS vein, and CS vein angiography showed VOM (Figure 1A).
2.4.2 The guiding catheter was advanced close to the proximal end of the VOM, then gently ran a guidewire to the distal end of the VOM (Figure 1B).
2.4.3 Approximately 3–6 ml of anhydrous ethanol was slowly injected into the distal end of the VOM through a dilated balloon catheter (Figure 1C).
2.4.4 Another 3–6 ml of anhydrous ethanol was slowly injected after the withdrawal of the balloon catheter to the proximal end of the VOM (Figure 1D).
2.4.5 When VOM angiography was performed, the ethanol-infused myocardium was stained with contrast agents (Figures 1C,D).

### 2.5. RFCA procedure

**Figure 2:**
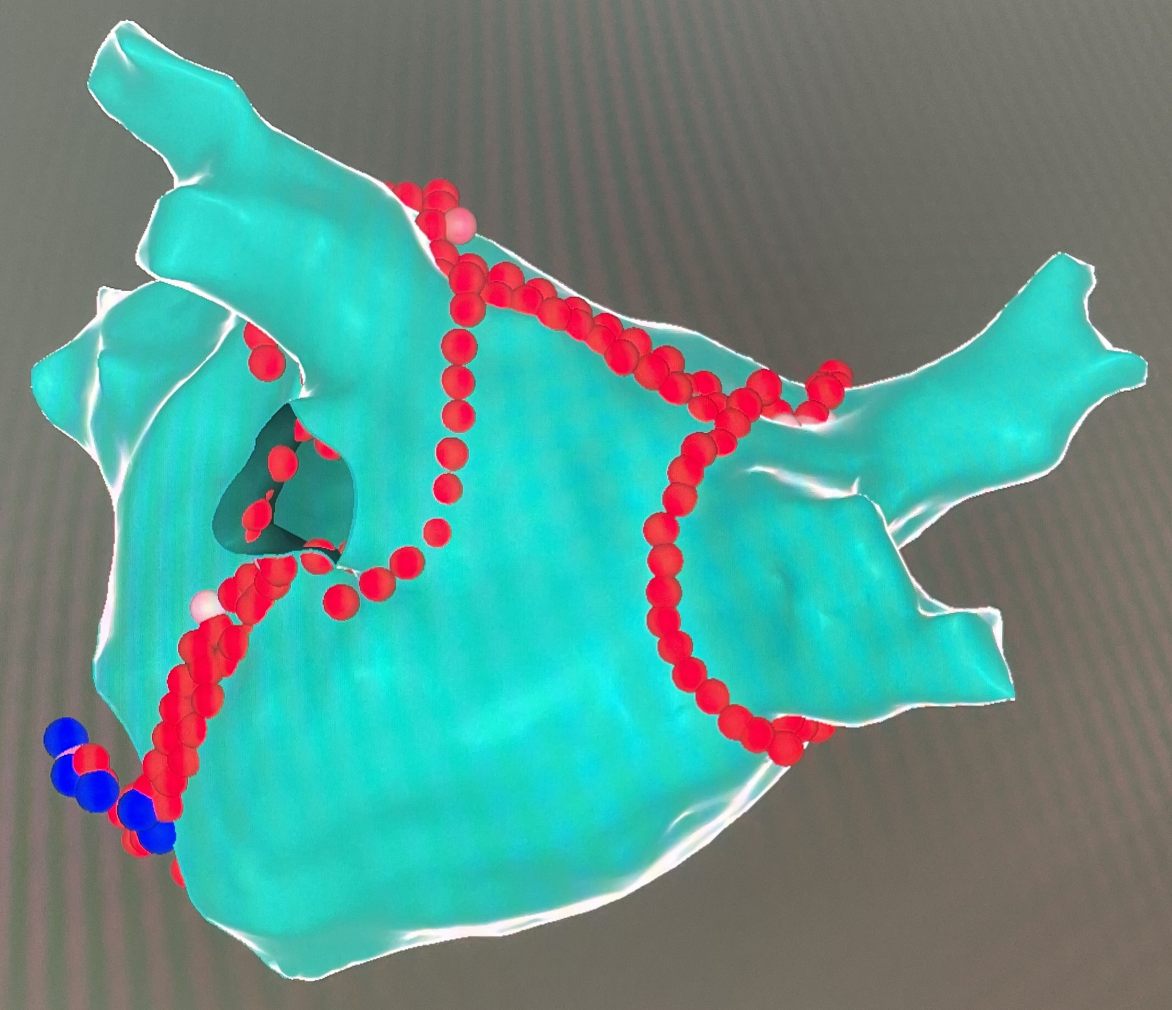
A typical LA electroanatomical map after CPVA and linear ablation in posterior-anterior views. The red dots indicate the site of endocardial ablation, and the blue dots indicate the site of CS vein ablation.

A high-density mapping catheter (Pentaray, Biosense Webster, Diamond Bar, CA, USA) was used to obtain an LA three-dimensional electroanatomical map. RFCA was performed using a thermocool ST SF uni-directional navigation catheter (Biosense Webster, Ciudad Juarez, Chihuahua, Mexico) at a power control mode (power 45W; saline flow rate 15 ml/min; temperature 43°C). CPVA and additional linear ablation were performed using an ablation index (AI)-guided technique (19). The target AI was: anterior wall 400–450; posterior wall 350; roof line 400; MI line 500; CTI line 450. CPVA ablation was accomplished per the “CLOSE” protocol, and PVI was established by the entrance and exit block (20). The LA roof line was created by point-to-point ablation connecting the lesions of the superior pulmonary veins. The conduction block was established by a shorter activation time at the lower than at the upper posterior wall of the LA during pacing at the LA appendage (21). Linear ablation of the posterior MI was accomplished according to procedures described in detail previously, and the bidirectional block was confirmed by differential pacing (22). Point-to-point ablation along the CTI from the ventricle to the atrium was conducted to complete the CTI line, and the bidirectional block was confirmed using a simplified differential pacing technique (23). The ablation within the CS vein was performed using a point-to-point course, with a power of 25W, a saline flow rate of 15 ml/min, and a target temperature of 43°C (24). We defined the CFAEs as continuous electrograms with a mean cycle length of less than 120 ms and conducted the ablation to eliminate all the areas identified with CFAEs altogether (25). Based on the LA roof line created previously, point-to-point ablation was performed to connect the lesions of the inferior pulmonary veins, thus achieving PWI. The exit block confirmed PWI as pacing from the posterior wall (26). After bilateral CPVA ablation, linear ablation of the roof and the MI was completed, and a typical LA electroanatomical map was obtained (Figure 2).

### 2.6. Postprocedure management

A proton pump inhibitor was administered until one month after the procedure to prevent esophageal injury. Anticoagulation therapy continues until at least three months after the procedure, after which it could be discontinued according to the CHA2DS2-VASc score. AADs were recommended for three months after the procedure, after which they could be stopped.

### 2.7. Follow-up

At recommended time points, including one month, three months, six months, and one year after the procedure, follow up through outpatient or telephone. An electrocardiogram (ECG) or Holter electrocardiogram (HOLTER) should be performed at the time point of follow-up or at any time when suspected arrhythmia-related symptoms occur. We defined the three months after the procedure as a blank period. AADs were preferred for relapse during the blank period, and a redo procedure could be performed for those who were ineffective or intolerant to drug therapy. Recurrence was defined as an episode of AF or AT longer than 30 s after the blank period or a redo procedure due to a recurrence during the blank period.

### 2.8. Statistical analysis

We represented the categorical variables as percentages and analyzed the data using Fisher exact test. Continuous standard distribution variables were described by mean ± standard deviation (mean ± SD), and Student's *t*-test was used for data analysis. A Kaplan–Meier curve represented survival without recurrent AF, and the difference between the two groups was assessed using the log-rank *p*-test. IBM SPSS 26.0 was used for statistical analysis, and *p* < 0.05 was considered statistically significant.

## 3. Results

### 3.1. Baseline characteristics

We enrolled 360 patients in the study, of which 141 were in the Elder group and 219 were in the Younger group. There was a significant age difference between the two groups, with a mean age difference of 15.5 years (69.6 ± 2.8 vs. 54.1 ± 5.6 years, *p* < 0.0001). The Elder group had fewer males (58.2% vs. 79.0%, *p* < 0.0001), a higher CHA2DS2-VASc score (2.71 ± 1.0 vs. 1.12 ± 0.9, *p* < 0.0001), and a higher baseline prevalence of hypertension (63.1% vs. 45.7%, *p* = 0.002), coronary atherosclerotic heart disease (30.5% vs. 15.1%, *p* = 0.0006), history of stroke (17.7% vs. 6.8%, *p* = 0.002). There were no significant differences in body mass index, LA diameter, time from first AF diagnosis, the prevalence of congestive heart failure, diabetes mellitus, and AAD usage between the two groups (Table 1).

**Table 1:** Baseline characteristics.

	Elder (*n* = 141)	Younger (*n* = 219)	*p*-value
Age, years	69.6 ± 2.8	54.1 ± 5.6	<0.0001
Male sex, *n* (%)	82 (58.2)	173 (79.0)	<0.0001
Body mass index	25.9 ± 3.1	26.3 ± 2.8	0.43
CHA2DS2-VASc score	2.71 ± 1.0	1.12 ± 0.9	<0.0001
LA diameter, mm	45.4 ± 4.2	45.0 ± 4.3	0.39
History of stroke, *n* (%)	25 (17.7)	15 (6.8)	0.002
Hypertension, *n* (%)	89 (63.1)	100 (45.7)	0.002
Diabetes mellitus, *n* (%)	18 (12.8)	26 (11.9)	0.87
Coronary atherosclerotic heart disease, *n* (%)	43 (30.5)	33 (15.1)	0.0006
Congestive heart failure, *n* (%)	16 (11.3)	34 (15.5)	0.28
Time from first AF diagnosis			
<1 year, *n* (%)	65 (46.1)	97 (44.3)	0.75
≥1 year, *n* (%)	76 (53.9)	122 (55.7)	0.75
AADs therapy			
Class Ⅰc, *n* (%)	6 (4.3)	19 (8.7)	0.14
Class Ⅱ, *n* (%)	28 (19.9)	55 (25.1)	0.31
Class Ⅲ, *n* (%)	71 (50.3)	106 (48.4)	0.75
Class Ⅳ, *n* (%)	17 (12.1)	29(13.2)	0.87

Data are shown as mean ± SD or absolute number and percentage (*n*%). LA, left atrium; AADs, antiarrhythmic drugs.

### 3.2. Procedural outcome

Procedural data were summarized in Table 2. The VOM-EI procedure was successfully conducted in 90.8% of the Elder and 88.6% of the Younger group. PVI was achieved in all patients, the LA roof block was achieved in 97.9% of the Elder and 98.6% of the Younger group, and the MI block was achieved in 93.6% of the Elder and 95.9% of the Younger group. The two groups were similar in total time of the procedure and the fluoroscopy, procedural and fluoroscopy time of VOM-EI and CPVA, LA roof, and MI ablation time. The successful rate of VOM-EI, PVI, LA roof block, and MI block was similar in the two groups. In addition, the two groups had no significant differences in the rate of CS vein, CTI, CFAE ablation, the rate of PWI, conversion to sinus rhythm, conversion to AT, and cardioversion.

**Table 2:** Procedural outcome.

	Elder (*n* = 141)	Younger (*n* = 219)	*p*-value
Total time of the procedure, min	159.5 ± 23.2	157.6 ± 23.0	0.71
Total time of the fluoroscopy, min	22.4 ± 6.4	22.5 ± 5.7	0.94
Successful VOM-EI	128 (90.8)	194 (88.6)	0.60
Procedural time of VOM-EI, min	22.4 ± 11.3	21.9 ± 11.1	0.68
Fluoroscopy time of VOM-EI, min	7.6 ± 4.1	7.2 ± 4.7	0.41
Successful PVI, *n* (%)	141 (100)	219 (100)	>0.9999
CPVA ablation time, min	45.6 ± 9.6	44.8 ± 8.1	0.69
LA roof block, *n* (%)	138 (97.9)	216 (98.6)	0.68
LA roof ablation time, min	6.5 ± 2.6	5.8 ± 1.9	0.15
MI block, *n* (%)	132 (93.6)	210 (95.9)	0.34
MI ablation time, min	18.0 ± 5.5	17.0 ± 6.3	0.53
CS vein ablation, *n* (%)	96 (68.1)	138 (63.0)	0.37
CTI ablation, *n* (%)	10 (7.1)	17 (7.8)	>0.9999
CFAE ablation, *n* (%)	15 (10.6)	18 (8.2)	0.46
PWI, *n* (%)	5 (3.5)	16 (7.3)	0.17
Convert to sinus rhythm, *n* (%)	12 (8.5)	21 (9.6)	0.85
Convert to AT, *n* (%)	22 (15.6)	28 (12.8)	0.53
Cardioversion, *n* (%)	107 (73.8)	170(77.6)	0.70

Data are shown as mean ± SD or absolute number and percentage (*n*%).

VOM-EI, VOM ethanol infusion; PVI, pulmonary vein isolation; CPVA, circumferential pulmonary vein antrum; LA, left atrium; MI, mitral isthmus; CS, coronary sinus; CTI, cavotricuspid isthmus; CFAE, complex fractionated atrial electrogram; PWI, posterior wall isolation; AT, atrial tachycardia.

### 3.3. Clinical outcome of one-year follow-up

**Figure 3:**
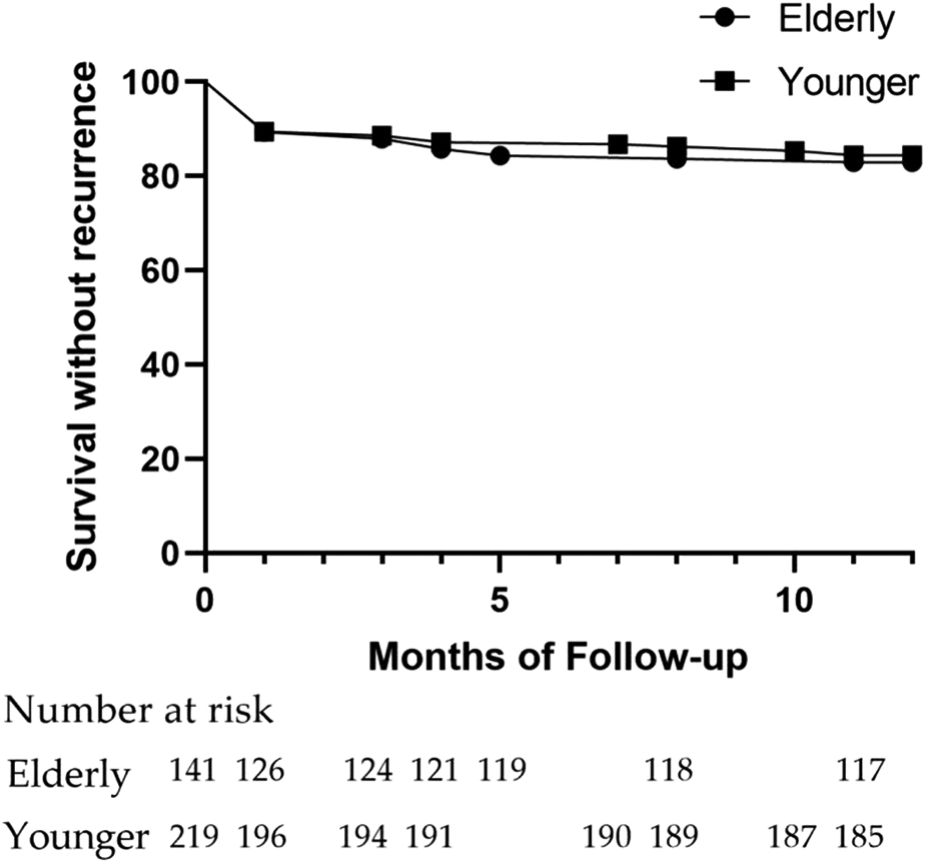
Survival curve without recurrence of AF one year after the procedure.

With no difference, freedom from AF/AT at one year was reached by 83.0% and 84.5% from the Elder and the Younger groups (Figure 3). The percentage of recurrence following the blank period (17.0% vs. 15.5%, *p* = 0.77), recurrence with AF or AT (12.8% vs. 12.8%, *p* > 0.9999; 9.2% vs. 5.0%, *p* = 0.13), the patients who underwent a redo procedure (7.8% vs. 5.0%, *p* = 0.37), AADs continuation post-procedure (34.0% vs. 33.3%, *p* = 0.91), and freedom from AF/AT off AADs (56.7% vs. 57.5%, *p* = 0.91) were similar between the two groups (Table 3).

**Table 3:** Clinical outcome of one-year follow-up.

	Elder (*n* = 141)	Younger (*n* = 219)	*p*-value
Freedom from AF/AT at one year, *n* (%)	117 (83.0)	185 (84.5)	0.77
Recurrence following the blank period, *n* (%)	24 (17.0)	34 (15.5)	0.77
Recurrence with AF, *n* (%)	18 (12.8)	28 (12.8)	>0.9999
Recurrence with AT, *n* (%)	13 (9.2)	11 (5.0)	0.13
Underwent a redo procedure, *n* (%)	11 (7.8)	11 (5.0)	0.37
AADs continuation post-procedure, *n* (%)	48 (34.0)	73 (33.3)	0.91
Freedom from AF/AT off AADs, *n* (%)	80 (56.7)	126 (57.5)	0.91

Data are shown as mean ± SD or absolute number and percentage (*n*%).

AF, atrial fibrillation; AT, atrial tachycardia; AADs, antiarrhythmic drugs.

### 3.4. Complications within 30 days postprocedure

The incidence of complications 30 days after the procedure, including acute cardiac tamponade (0.71% vs. 0.46%, *p* > 0.9999), pericardial effusion without pericardiocentesis (0.71% vs. 1.83%, *p* = 0.65), hematoma (one patient in the Elder group), arteriovenous fistula (one patient in the Elder group), and stroke (0.71% vs. 0.91%, *p* > 0.9999), was not significantly different between the two groups (Table 4).

**Table 4:** Complications within 30 days postprocedure.

	Elder (*n* = 141)	Younger (*n* = 219)	*p*-value
Acute cardiac tamponade, *n* (%)	1 (0.71)	1 (0.46)	>0.9999
Pericardial effusion without pericardiocentesis, *n* (%)	1 (0.71)	4 (1.83)	0.65
Hematoma, *n* (%)	1 (0.71)		
Arteriovenous fistula, *n* (%)	1 (0.71)		
Stroke, *n* (%)	1 (0.71)	2 (0.91)	>0.9999
Total complications, *n* (%)	5 (3.55)	7 (3.2)	>0.9999

Data are shown as mean ± SD or absolute number and percentage (*n*%).

## 4. Discussion

### 4.1. Main findings

The VOM-EI procedure was successfully performed in 90.8% of patients from the Elder group and 88.6% from the Younger groups, suggesting its feasibility as an adjunctive strategy for AF ablation. The one-year AF/AT-free survival rate in the Elder and the Younger groups reached 83.0% and 84.5%, respectively, suggesting the efficacy of VOM-EI combined with RFCA for AF. The VOM-EI combined RFCA strategy was associated with a lower complication incidence within 30 days after the procedure, suggesting the safety of the strategy. There was no significant difference in procedure nor clinical outcomes between the two groups, implying that VOM-EI combined with RFCA could become a recommendable strategy for treating AF in elderly patients.

### 4.2. Feasibility of the VOM-EI with RFCA strategy

The VOM contains triggers and sympathetic and parasympathetic nerves and is, therefore, implicated in the pathogenesis of AF (27). In addition, the anatomical location of VOM is consistent with that of the MI. It plays a crucial role in MI-dependent atrial flutter, the leading cause of ablation failure for AF (28). The VOM can be ablated by ethanol infusion to eliminate triggers for AF and facilitate MI ablation (15). In the VENUS clinical trial, the VOM-EI combined with RFCA improved sinus rhythm maintenance one year after the procedure in patients with PsAF. The VOM-EI procedure needed approximately 42.5 min on average and had an overall success rate of 83.7% (16). In our investigation, 360 patients completed the VOM-EI procedure and needed approximately 22 min on average, with an overall success rate of roughly 90%. In our study, 38 patients did not complete the VOM-EI procedure, 13 from the elder group and 25 from the younger group (9.2% and 11.4% of the total number of included cases, respectively). In these cases, two from the elder group and three from the younger group, the failure was due to the difficulty of introducing the guidewire or balloon to the VOM. The failure of the remaining 33 cases was due to the absence of VOM after repeated CS vein angiography. The VOM-EI procedure in our study required less time and had a higher success rate, suggesting that additional implementation of VOM-EI during catheter ablation for AF is feasible.

### 4.3. Efficacy of the VOM-EI with RFCA strategy

PVI is an established AF treatment but is inadequate for PsAF (5–7). Earlier studies have suggested that linear and (or) CFAE ablation, in addition to PVI, failed to improve the ablation outcome (11–14). However, the recent EARNEST-PVI trial suggested that PVI plus linear and (or) CFAE ablation was promising to improve clinical efficacy (10). In the trial, 78.3% of patients in the PVI plus linear and (or) CFAE ablation group had no recurrence of AF one year after a single procedure (10). VOM-EI has become an established ablation strategy for AF in recent years (15). The VENUS clinical trial evaluated the efficacy of CA plus VOM-EI in patients with PsAF and achieved a positive result. CA plus the VOM-EI strategy increased freedom from AF recurrence compared to PVI alone one year after a single procedure (49.2% vs. 38%) (16). Disappointingly, both ablation strategies had low success rates. More recently, another clinical retrospective study evaluated the benefit of additional VOM-EI in patients with PsAF. The adjunctive VOM-EI strategy beyond the PVI plus linear ablation improved freedom from AF recurrence compared with PVI plus linear ablation one year after the procedure (87.9% vs. 64.8%). In addition, the VOM-EI strategy was associated with a higher rate of MI block (95.5% vs. 80.8%) (17). In our study, one year after the procedure, freedom from AF recurrence reached 83.0% and 84.5% in the Elder and Younger groups, respectively. Moreover, the success rate of MI block reached 93.6% and 95.9% in the Elder and Younger groups, respectively. These findings suggested that adjunctive VOM-EI could become an effective ablation strategy, and the benefit might be primarily due to its facilitation of MI ablation.

### 4.4. Safety of the VOM-EI with RFCA strategy

Total complications within 30 days postprocedure were five from the Elder group and seven from the Younger group. Reviewing the ablation procedures concluded that the reason for acute pericardial tamponade in two cases was that the AI far exceeded the target value. Pericardial effusion occurred in five patients and was absorbed by itself after 1–3 days of observation. We speculated that the cause of pericardial effusion may be pericarditis caused by ablation injury. Three strokes were reported in our study, of which two (one in the elder group and one in the younger group) were due to severe cerebral artery stenosis and perioperative blood pressure fluctuation. Another stroke occurred in the younger group due to intraoperative microthromboembolism. Of the three stroke patients, one underwent an emergency interventional thrombectomy procedure, and the other two went into remission with medication. The overall incidence of complications was 3.55% in the Elder group and 3.2% in the Younger group. Both groups had low complication rates, and no deaths related to the procedure occurred. These results suggested that VOM-EI combined with RFCA could be a safe strategy for PsAF in elderly patients.

### 4.5. VOM-EI with RFCA strategy for elderly patients

Several studies have suggested that the recurrence and complication rates of elderly patients with AF after ablation are similar to those of non-elderly patients (29–31). A recent meta-analysis indicated that recurrence and complication rates after AF ablation were higher in people ≥60 years than those younger than 60, except those aged ≥5 years (32). In these studies evaluating the efficacy and safety of AF ablation in elderly patients, the types of AF included and the different ablation strategies may be the main reasons for the inconsistent conclusions of the studies. The traditional definition of an older adult is ≥65 years old; in recent years, researchers have defined an older adult as ≥75 (33). At our electrophysiology Center, patients with AF ≥ 75 years of age have a low rate of catheter ablation. Therefore, in our study, older patients with AF were still defined by age ≥65. VOM-EI combined with RFCA has been a widely accepted AF ablation strategy in recent years, and its efficacy and safety are still uncertain in the elderly population. In our study, the efficacy and safety of VOM-EI combined with RFCA for treating PsAF were similar in patients ≥65 years compared with those younger than 65. With the aging of the population, more and more patients ≥75 years of age are receiving VOM-EI combined with RFCA as a therapeutic strategy, and its efficacy and safety need to be evaluated in further studies.

### 4.6. Limitations

There were several limitations in the study. First, this is only a retrospective observational study; Second, we might miss some recurrence of AF due to the lack of continuous ECG recording; Third, due to the limited sample size, the study lacked a separate comparative study for patients over 75 years old. Finally, the study follow-up period was one year, and more extended follow-up periods should be completed.

## 5. Conclusions

The adjunctive VOM-EI during RFCA for PsAF was associated with above 80% of one-year AF/AT-free survival and above 90% of MI block. In patients ≥65 years of age, one-year freedom from AF and complications incidence showed no difference compared to patients <65. These findings suggested that VOM-EI combined with RFCA could become a recommended strategy for treating PsAF in elderly patients.

## Data Availability

The original contributions presented in the study are included in the article/supplementary material, further inquiries can be directed to the corresponding author.
